# Machine Learning Framework for Multi-Endpoint Quantum Dot Toxicity Prediction with Organoid Validation and Drug Target Discovery

**DOI:** 10.3390/toxics13110967

**Published:** 2025-11-10

**Authors:** Jiafu Yang, Dayu Hu, Pengcheng Xing, Yikai Zhang, Zongjian Ye, Kehan Liu, Jieyi Xia, Jing He, Yijing Qian, Tianshu Wu

**Affiliations:** Key Laboratory of Environmental Medicine and Engineering of Ministry of Education, School of Public Health, Southeast University, Nanjing 210009, China; jiafuyang0825@163.com (J.Y.); 220234068@seu.edu.cn (D.H.); 230249302@seu.edu.cn (P.X.); 230250191@seu.edu.cn (Y.Z.); 220234071@seu.edu.cn (Z.Y.); 220234041@seu.edu.cn (K.L.); 220244301@seu.edu.cn (J.X.); 220244222@seu.edu.cn (J.H.); 213211114@seu.edu.cn (Y.Q.)

**Keywords:** nanotoxicology, artificial intelligence, molecular docking, network pharmacology, organoids

## Abstract

Quantum dots (QDs) possess unique optical and electronic properties, enabling wide applications in biomedicine and optoelectronics, but their nanoscale size and surface chemistry could pose potential toxicity risks. This study established a systematic, multi-endpoint framework for QD toxicity assessment. Physicochemical properties of various QDs and their multiple toxicity endpoints, including cell death, inflammation, and oxidative stress, were collected to build machine learning models (RF, XGBoost, KNN, SVM). The predictive toxic effects were then validated based on the brain organoid. Shapley Additive exPlanations (SHAP) analysis revealed that exposure dose and particle size were key cross-model drivers, while zeta potential and optical properties differentially affected specific toxicity endpoints. Integration of GEO-derived differentially expressed genes with protein–protein interaction networks and molecular docking showed that the proteasome inhibitor Carfilzomib is an efficient interventive drug because of its strongest binding to core targets. In this study, the framework of prediction, validation and intervention effectively evaluated multi-endpoint QD toxicity and provided a systematic approach for safety assessments and strategy developments of nanomaterials.

## 1. Introduction

Quantum Dots (QDs) are semiconductor nanomaterials exhibiting quantum confinement effects [1]. They were first synthesized in a glass matrix by Soviet physicist Alexei Ekimov in 1981. Typically, the size of QDs ranges from 1 to 10 nm [2]. Due to the three-dimensional spatial confinement of electron and hole movements, they form discrete, atom-like energy level structures, often referred to as “artificial atoms” [3,4,5]. Since the 1990s, QDs have demonstrated broad application prospects in fields such as fluorescence labeling, biological imaging, optoelectronic devices, and medical diagnostics, owing to their unique optical and electronic properties [6,7,8,9,10]. In terms of the market, the demand for QDs continues to grow. For example, the global QD healthcare market was estimated at USD 4 billion in 2021 and is projected to reach USD 8.6 billion over the next five years [11].

However, the nanoscale dimensions and surface chemical properties of QDs also pose potential biotoxicity risks. Numerous studies have indicated that exposure to QDs may trigger key toxicity endpoints, including oxidative stress, inflammatory responses, and cell death [12,13,14,15,16], For example, nitrogen-doped graphene quantum dots can disrupt intracellular calcium homeostasis by activating two calcium channels, thereby inducing ferroptosis and inflammatory responses in mouse hippocampal tissues and cultured microglial cells [14]. Research by Liang, Chen, and others has revealed that exposure to QDs can cause nervous system damage in Caenorhabditis elegans, accompanied by abnormal protein aggregation and neuronal injury [12,17]. Furthermore, exposure to QDs through various routes (e.g., skin contact, inhalation, gavage, and intravenous injection) may pose potential health hazards to organisms, including neurotoxicity, pulmonary toxicity, renal toxicity, hepatotoxicity, and other health risks [15,18,19,20,21,22].

The toxicity of QDs is complexly regulated by multiple factors. Previous experimental studies have shown that exposure dose, duration, and particularly physicochemical properties play crucial roles [23,24,25]. The composition of QDs, such as core materials (e.g., cadmium, selenium, zinc, graphene) [26,27,28,29], as well as their shape, particle size, charge, surface modification, and solubility, collectively influence their interactions with biomolecules and thereby determine their biological effects [24,30,31,32]. In recent years, with the advancement of big data and artificial intelligence, machine learning methods have been introduced into nanotoxicology research. By integrating the physicochemical properties of QDs with existing toxicity data, these methods can predict multi-endpoint toxicity and identify potential key influencing factors, significantly enhancing the efficiency and accuracy of toxicity assessment [33,34,35].

**Figure 1:**
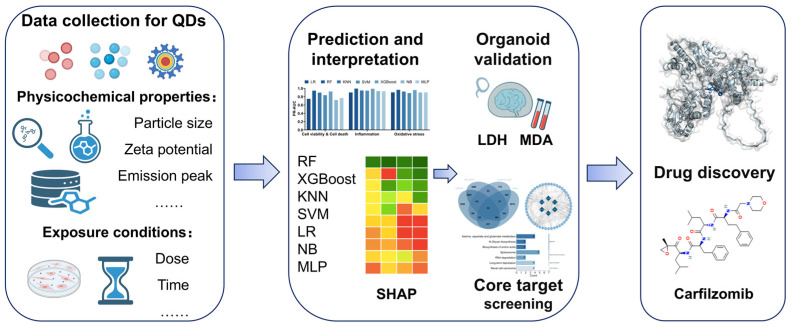
Machine learning framework for multi-endpoint quantum dot (QD) toxicity prediction with organoid validation and drug target discovery. This schematic illustrates the overall workflow integrating data preprocessing, feature selection, model training and validation, SHAP-based feature interpretation, organoid validation, and drug target prediction. Abbreviations: SHAP, Shapley Additive exPlanations; MDA, malondialdehyde; LDH, lactate dehydrogenase; RF, random forest; XGB, eXtreme Gradient Boosting; SVM, support vector machine; LR, logistic regression; NB, naïve Bayes; MLP, multi-layer perceptron; KNN, k-nearest neighbors.

As a prevalent technique, machine learning (ML) plays a more significant role in various domains and diseases [36,37,38]. Despite the potential of machine learning in nanotoxicology, most current studies focus on single toxicity endpoints and lack experimental validation [39,40,41,42,43]. Existing in vitro and in vivo toxicity studies are often fragmented and have high experimental costs, making systematic predictions challenging. Given the potential biotoxicity risks associated with QDs, conducting systematic and comprehensive toxicity assessments is crucial for ensuring their safety in biomedical and industrial applications. Therefore, this study collected physicochemical properties and multiple toxicity endpoint indicators of QDs from existing research data to construct a machine learning-based multi-endpoint prediction system. The prediction results were validated through organoid experiments. Additionally, potential target genes were screened using GEO data, and network pharmacology analysis was conducted to identify potential intervention drugs, followed by molecular docking analysis to evaluate potential pharmacological intervention strategies (Figure 1). This study established a multi-module integrated framework of prediction–validation–intervention, providing a systematic approach for QD toxicity assessment and intervention. It also offers theoretical foundations and practical guidance for developing potential interventions against QD-induced damage, holding significant scientific research value and application prospects.

## 2. Materials and Methods

### 2.1. Data Collection, Imputation, and Multi-Model Training

Data on the physicochemical properties and toxicological outcomes of QDs were collected from 102 published reports. After screening for completeness and quality, 40 studies were finally included, providing 306 valid records from the 646 initially extracted. To control data quality, we set a maximum missing rate of 40% for each variable. This threshold was chosen as a balance between data completeness and reliability, since a stricter criterion would greatly reduce the dataset size, while a looser one could introduce excessive uncertainty. Samples or variables exceeding this threshold were excluded. For the remaining data, missing feature values were imputed using the K-nearest neighbors (KNN, k = 5) method to preserve the overall structure and distribution. Because the three biological endpoints (cell viability/death, inflammation, and oxidative stress) were binary, samples with known outcomes were used to train a Random Forest classifier, which was then applied to predict missing outcome values. The final dataset included ten physicochemical and exposure-related features (Size (H_2_O-DLS), Size (TEM), Excitation peak, Emission peak, Zeta potential, Exposure duration, Exposure dose, Dose unit, Exposure route, and Post-exposure period) and three toxicity outcomes (Cell viability/death, Inflammation, and Oxidative stress).

**Figure 2:**
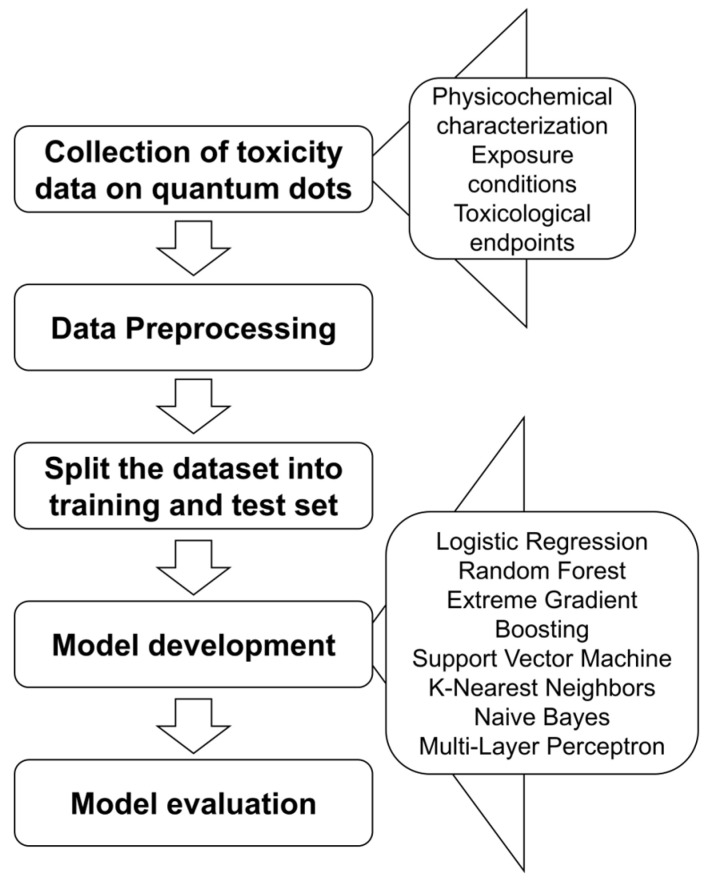
Workflow for developing seven machine learning models to predict quantum dot-induced toxicities based on physicochemical properties.

The complete dataset was divided into training and testing sets using a 70/30 stratified split to maintain balanced class proportions. Given the limited sample size, we did not perform repeated random splits or multi-fold cross-validation for overall model evaluation. Instead, 3-fold cross-validation within the RandomizedSearchCV framework was used for hyperparameter tuning to ensure that model selection remained stable across folds. Seven supervised machine learning algorithms were then trained and compared, including Random Forest (RF), Extreme Gradient Boosting (XGBoost), K-Nearest Neighbors (KNN), Support Vector Machine (SVM), Naive Bayes (NB), Logistic Regression (LR), and Multi-Layer Perceptron (MLP). The complete data processing and modeling workflow is summarized in Figure 2, and all features and outcome variables used in the analysis are listed in Table S1.

### 2.2. Hyperparameter Tuning of Machine Learning Models

#### 2.2.1. K-Nearest Neighbors

For the KNN model, hyperparameters including the number of neighbors, weighting scheme, and distance metric were optimized using RandomizedSearchCV with 3-fold cross-validation, with ROC-AUC as the evaluation metric.

#### 2.2.2. Logistic Regression

For the LR model, hyperparameter optimization was performed using RandomizedSearchCV with 3-fold cross-validation. The search space included the regularization strength C (0.01–10, continuous range), penalty type (L1 or L2), and solver (liblinear or lbfgs). Model performance was evaluated using the ROC-AUC score, and the optimal parameters were selected accordingly.

#### 2.2.3. Naive Bayes

For the NB model, we used the GaussianNB algorithm. Since it has few hyperparameters, with var_smoothing being the main one, we tuned it using 3-fold cross-validation and selected the setting that achieved the best ROC-AUC performance.

#### 2.2.4. Random Forest

For the RF model, hyperparameter optimization was performed using RandomizedSearchCV with 3-fold cross-validation. The search space included the number of trees (50–200), maximum depth (3–15), minimum samples for split (2–10), and minimum samples per leaf (1–5). The optimal model was selected based on the best ROC-AUC score.

#### 2.2.5. Support Vector Machine

For the SVM model, hyperparameter optimization was carried out using RandomizedSearchCV with 3-fold cross-validation. The search space covered the regularization parameter C (0.1–10) and the kernel coefficient gamma (exponential distribution, scale = 0.1), with the RBF kernel fixed. The optimal configuration was determined based on the highest ROC-AUC score.

#### 2.2.6. Extreme Gradient Boosting

For the XGBoost model, hyperparameter optimization was conducted using RandomizedSearchCV with 3-fold cross-validation. The search space included the number of trees, maximum depth, learning rate, subsample ratio, column sampling ratio, and gamma. The optimal model was selected based on the highest ROC-AUC score and was subsequently used for prediction and feature importance analysis.

#### 2.2.7. Multi-Layer Perceptron

For the MLP model, hyperparameter optimization was conducted using RandomizedSearchCV with 3-fold cross-validation. The search space included the hidden layer structure, activation function, regularization strength (alpha), and initial learning rate. The optimal model was selected based on the highest ROC-AUC score and was subsequently used for prediction and feature importance analysis.

### 2.3. Machine Learning Model Evaluation

A standardized workflow was applied for all machine learning models in this study. For each outcome, the dataset was stratified to maintain the original class distribution and then split into training and test sets. Continuous features were standardized to remove scale differences, while categorical features were one-hot encoded to allow model input. Each model was trained on the training set, and hyperparameters were optimized using randomized search combined with cross-validation to select the best-performing configuration. Model performance was then evaluated on the test set using a comprehensive set of metrics, including accuracy, sensitivity, specificity, precision, F1 score, ROC-AUC, and PR-AUC, providing a robust assessment of predictive performance.

### 2.4. SHAP Feature Importance Analysis

SHAP values were calculated for all seven machine learning models to interpret their predictions. The training data were used as the background for computing SHAP values on the test set, ensuring that feature contributions were assessed relative to the original data distribution. For each feature, the mean absolute SHAP value was computed to quantify its impact, with continuous and categorical features handled appropriately. The results were visualized as bar plots, highlighting the relative importance of each feature for the different outcomes.

### 2.5. The Physicochemical Characterizations of QDs

CQDs, N-GQDs, and CdTe QDs were purchased from XFNANO Materials Tech Co., Ltd. (Nanjing, China; http://www.xfnano.com, accessed on 11 October 2024) and characterized for their physicochemical properties prior to use. The structural morphology of the QDs was examined by high-resolution transmission electron microscopy (HR-TEM, JEM-2100, JEOL, Tokyo, Japan), while their fluorescence lifetimes were measured using a time-resolved fluorescence spectrometer (Edinburgh FLSP980, UK). Hydrodynamic size and surface ζ-potential were determined with a Malvern Zetasizer Nano ZS (Zetasizer Nano-ZS90, Malvern Instruments, Worcestershire, UK). A summary of the physicochemical properties of the CQDs is provided in Table S2.

### 2.6. Generation and Quantum Dot Exposure of Human Brain Organoids

Human brain organoids were generated from human embryonic stem cells (H9 line) through stepwise induction. Briefly, cells were maintained in Essential 8 medium, and when they reached approximately 70% confluency, they were dissociated with Dispase to form embryoid bodies (EBs). The EBs were cultured in neural induction medium (NIM) supplemented with SB431542 and DMH1 for 7 days, then embedded in Matrigel to promote neuroepithelial formation, followed by culture in NIM without small-molecule inhibitors. Over time, the organoids gradually developed ventricular-like structures and neuroepithelial features, and by day 30, they exhibited stable morphological and differentiation characteristics and could be maintained in long-term culture. To evaluate the toxic effects of quantum dots, organoids at days 10, 20, and 30 were exposed for 24 h, after which lactate dehydrogenase (LDH) release and malondialdehyde (MDA) levels were measured to assess cell damage and oxidative stress. The assays for LDH and MDA were performed according to the manufacturer’s instructions (Solarbio, Beijing, China).

### 2.7. Identification and Functional Analysis of Potential Target Genes in Response to QD Exposure

Differentially expressed genes (DEGs) were obtained from GEO datasets and intersected to identify potential target genes. Gene Ontology (GO) and Kyoto Encyclopedia of Genes and Genomes (KEGG) pathway enrichment analyses of these potential targets were performed using the Metascape platform (https://metascape.org, accessed on 26 August 2025). Protein–protein interaction (PPI) networks were constructed and analyzed using Cytoscape 3.10 to identify key hub genes. Six core target genes were selected based on network topology, and GO and KEGG enrichment analyses were further conducted on these core genes using Metascape.

### 2.8. Screening of Potential Therapeutics for QD-Induced Toxicity

Based on the six key target genes identified in this study, potential therapeutic agents were screened using the DGIdb Drug Targets 2024 dataset and the MAGMA Drugs and Diseases dataset via the Enrichr platform. Selected candidate drugs were subjected to molecular docking with the target proteins, and binding free energies were calculated using AutoDock Vina 1.2.7. Protein–ligand interactions, including hydrogen bond numbers and interaction patterns, were analyzed using the Protein–Ligand Interaction Profiler (PLIP) platform. Three-dimensional visualization of the complexes and annotation of binding sites, hydrogen bonds, and binding energies were performed using PyMol. It should be noted that AutoDock Vina 1.2.7 does not support boron parameterization; therefore, for Bortezomib, the boron atom was replaced with a carbon atom during docking. This approach is scientifically reasonable, as it does not significantly alter the overall molecular scaffold or binding mode, allowing the docking process to proceed smoothly and providing a reasonable prediction of ligand–protein binding trends.

### 2.9. Data Analysis and Statistics

Data are expressed as mean ± standard deviation (SD). Statistical analyses were performed using GraphPad Prism 9.0. Differences among groups were evaluated by one-way ANOVA, followed by Dunnett’s post hoc test for multiple comparisons. A *p*-value < 0.05 was considered statistically significant, with *p*-value < 0.01 and *p*-value < 0.001 indicating high and very high significance, respectively.

## 3. Results

### 3.1. Performance Comparison of Seven Machine Learning Models in Predicting Quantum Dot-Induced Cell Viability, Inflammation, and Oxidative Stress

**Figure 3:**
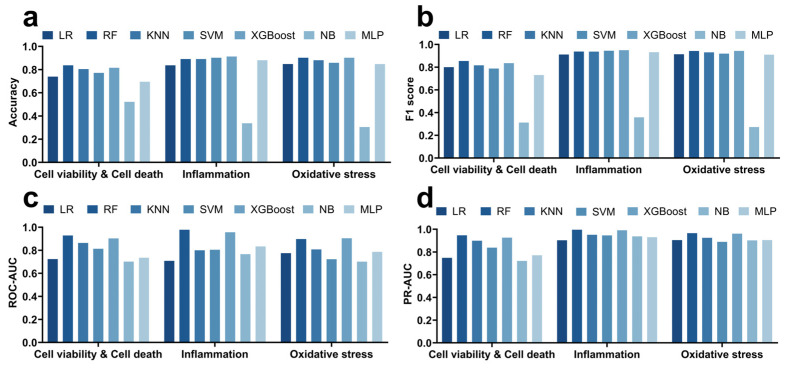
Comparison of seven machine learning models (RF, XGB, KNN, SVM, LR, NB, and MLP) in predicting quantum dot-induced cell death, inflammation, and oxidative stress based on accuracy, F1 score (**a**,**b**), ROC-AUC (**c**), and PR-AUC (**d**). Other evaluation metrics are provided in Table S3.

In this study, we systematically compared the performance of seven mainstream machine learning models (RF, KNN, XGBoost, SVM, LR, NB, and MLP) in predicting three toxicity endpoints induced by quantum dots: cell death, inflammatory response, and oxidative stress (Table S3, Figure 3). For the prediction of cell viability and cell death, the RF model demonstrated the best performance, achieving an accuracy of 0.837, an F1 score of 0.854, an ROC-AUC of 0.928 (Figure 3c), and a PR-AUC of 0.947 (Figure 3d), indicating a high overall predictive capability. The KNN, XGBoost, and SVM models also exhibited good performance, with F1 scores of 0.816, 0.835, and 0.788, respectively, although there was a trade-off between sensitivity and specificity. For example, the NB model had a sensitivity of only 0.196 but a specificity as high as 0.927, suggesting its tendency to predict negative samples. Overall, RF and XGBoost outperformed the other models in terms of comprehensive performance metrics.

In the prediction of inflammatory response (Table S3, Figure 3), the XGBoost model performed the best, achieving an accuracy of 0.913, an F1 score of 0.949, a PR-AUC of 0.991, and an ROC-AUC of 0.956. The SVM and RF models also performed well, with F1 scores of 0.945 and 0.938, respectively. However, the NB model showed significantly lower accuracy and F1 score (0.337 and 0.358, respectively), indicating its insufficient ability to predict positive samples. Notably, the sensitivity of most models was close to or reached 1, suggesting their good ability to capture positive inflammatory samples, but the specificity metrics were relatively low, indicating limited predictive capability for negative samples.

In the prediction of oxidative stress (Table S3, Figure 3), the RF and XGBoost models also performed outstandingly, both achieving an accuracy of 0.902, with F1 scores of 0.942 and 0.943, respectively, and PR-AUCs of 0.966 and 0.962, respectively, demonstrating high discriminative ability between positive and negative samples. The KNN model followed closely, while the NB model, although exhibiting high specificity (0.941), had a sensitivity of only 0.16, resulting in poor overall performance. Overall, the RF and XGBoost models maintained high robustness in predicting the three toxicity endpoints, particularly excelling in capturing positive samples.

Comprehensive analysis revealed that the RF and XGBoost models demonstrated the best overall performance in predicting cell death, inflammatory response, and oxidative stress, leading in terms of accuracy, F1 score, ROC-AUC, and PR-AUC. The KNN and SVM models performed secondarily, while the MLP model showed moderate performance. Although the NB model exhibited performance in some metrics, its overall accuracy and F1 score were significantly lower, suggesting its unsuitability for comprehensive toxicity prediction in this dataset.

### 3.2. SHAP Analysis of the Physicochemical Properties and Exposure Conditions of Quantum Dots Under Different Toxicological Outcomes

**Figure 4:**
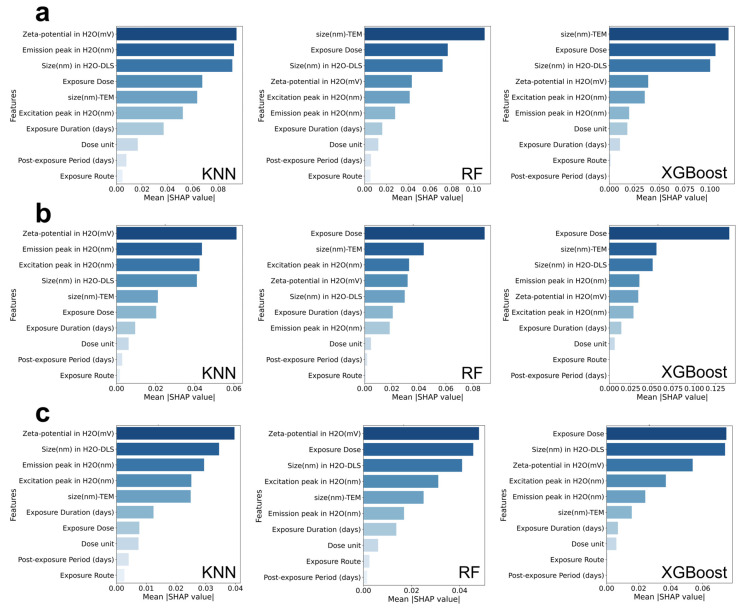
Mean SHAP values of the top three performing models for predicting quantum dot-induced cell death (**a**), inflammation (**b**), and oxidative stress (**c**).

In this section, we conducted SHAP value analysis on the relevant features of each model (Figure 4 and Figure S1). In the prediction of cell death induced by quantum dots (Figure 4a), the SHAP value analyses of the three best-performing models (KNN, RF, and XGBoost) all indicated that characterization parameters and exposure conditions jointly drove the prediction results. Specifically, the KNN model revealed that Zeta potential, emission peak, and hydrodynamic diameter (H_2_O-DLS) in aqueous solution made the greatest contributions to the model’s output; the RF model emphasized the importance of particle size measured by transmission electron microscopy (TEM) and exposure dose; the XGBoost model further highlighted the dominant roles of exposure dose and particle size (both TEM and H_2_O-DLS). These results indicate that cell death is closely associated with both the physicochemical properties of the quantum dots and the exposure dose, suggesting that variations in size, surface charge, or composition may significantly influence their cytotoxic effects.

In the prediction of inflammatory outcomes (Figure 4b), the KNN model similarly underscored the importance of Zeta potential and emission peak in aqueous solution; the RF model highlighted the contributions of exposure dose and particle size (TEM); and the XGBoost model reaffirmed that exposure dose was the most significant driving factor, followed by particle size and optical properties (excitation peak, emission peak). These results indicate that the inflammatory response is primarily influenced by the exposure dose, while physicochemical properties such as particle size and surface charge also play important regulatory roles. This suggests that both the amount of exposure and the intrinsic characteristics of the particles contribute to the observed pro-inflammatory effects.

In the prediction of oxidative stress (Figure 4c), the KNN model results showed that Zeta potential and hydrodynamic diameter were the primary influencing factors; the RF model emphasized the roles of exposure dose and Zeta potential; and the XGBoost model results further stressed the decisive contribution of exposure dose, accompanied by the auxiliary roles of particle size and optical characteristics. Overall, the occurrence of oxidative stress is closely related to the surface charge of quantum dots and the exposure dose.

In summary, the predictions for all three outcomes consistently highlighted exposure dose and particle size as major contributing factors across different models and toxicity endpoints. Meanwhile, Zeta potential and optical properties appeared to influence specific outcomes in distinct ways. These findings suggest that both the physicochemical characteristics of quantum dots and the exposure conditions together shape their observed biological toxicity.

### 3.3. Toxic Effects of Different Types of Quantum Dots in Brain Organoids and Machine Learning Validation

**Figure 5:**
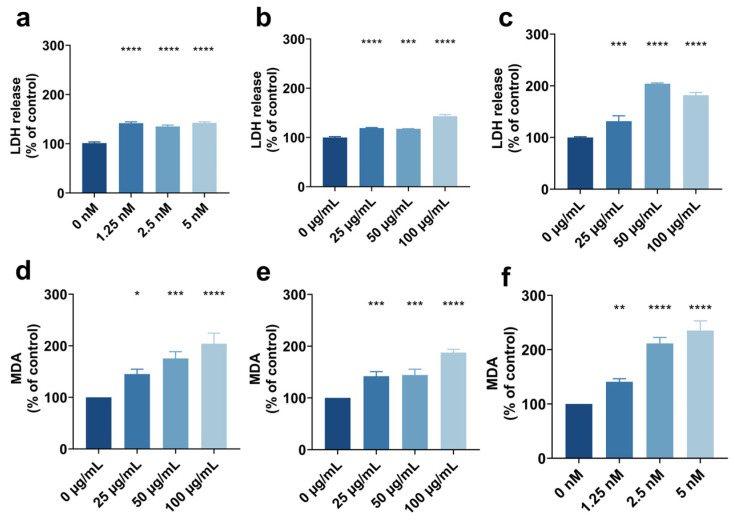
LDH release in brain organoids cultured for 10 days induced by CdTe QDs (**a**), for 20 days induced by CQDs (**b**), and for 30 days induced by N-GQDs (**c**); MDA content elevation in 30-day cultured brain organoids following exposure to CQDs (**d**), N-GQDs (**e**), and CdTe QDs (**f**). A *p*-value < 0.05 was considered statistically significant (*), with *p*-value < 0.01 (**), *p*-value < 0.001 (***), and *p*-value < 0.0001 (****) indicating progressively stronger levels of significance.

To assess the toxic effects of different types of QDs on brain organoids, we measured LDH release and MDA production. We found that CdTe QDs markedly increased LDH release in organoids cultured for 10 days (Figure 5a). Moreover, LDH levels rose in a clear dose-dependent manner as the concentration increased from 1.25 to 5 nM, indicating that higher exposure led to more pronounced cell damage. In brain organoids cultured for 20 days, exposure to CQDs also significantly promoted LDH release (Figure 5b), showing a concentration-dependent enhancement effect within the range of 25–100 μg/mL. Furthermore, in brain organoids cultured for 30 days, exposure to N-GQDs also led to a significant increase in LDH (Figure 5c), indicating that different types of QDs can all induce cell damage in brain organoids.

In addition to cell death markers, we assessed lipid peroxidation levels. The results demonstrated that CQDs, N-GQDs, and CdTe QDs could all significantly elevate MDA levels (Figure 5d–f). Among them, CQDs caused the maximum increase in MDA at 100 μg/mL; N-GQDs exhibited a strong pro-oxidative effect across all dose groups; while exposure to CdTe QDs resulted in a significant increase in MDA within the range of 2.5–5 nM. Overall, different types of QDs could induce marked cell damage and oxidative stress in brain organoids, with dose-dependent variations.

To further validate the reliability of the machine learning results, we systematically compared the experimental results of the three types of QDs with the predictive results of the KNN, RF, and XGBoost models (Table S4). The results showed that all three models were highly consistent with the experimental results in predicting toxicity in terms of cell viability and oxidative stress. Specifically, CQDs and N-GQDs were accurately predicted by all models to exhibit cytotoxic and oxidative stress effects; some predictive results for CdTe QDs showed slight discrepancies with the experimental results, but overall, they were in agreement. These findings indicate that machine learning models possess high accuracy and reliability in predicting QD toxicity and can corroborate actual experimental data from brain organoids.

### 3.4. Differential Gene Expression Screening and Functional Enrichment Analysis Induced by Quantum Dots

Subsequently, we screened for DEGs in four GEO datasets, with the results depicted in volcano plots (Figure S2a–d). In GSE96720, 5317 significantly upregulated genes and 6226 downregulated genes were identified; in GSE159776, there were 3418 upregulated genes and 3395 downregulated genes; in GSE89756 (3-day treatment), 1033 upregulated genes and 854 downregulated genes were detected; while in GSE89756 (21-day treatment), 1991 upregulated genes and 1674 downregulated genes were found. These findings indicate that different quantum dot treatments can induce significant gene expression changes under various experimental conditions.

**Figure 6:**
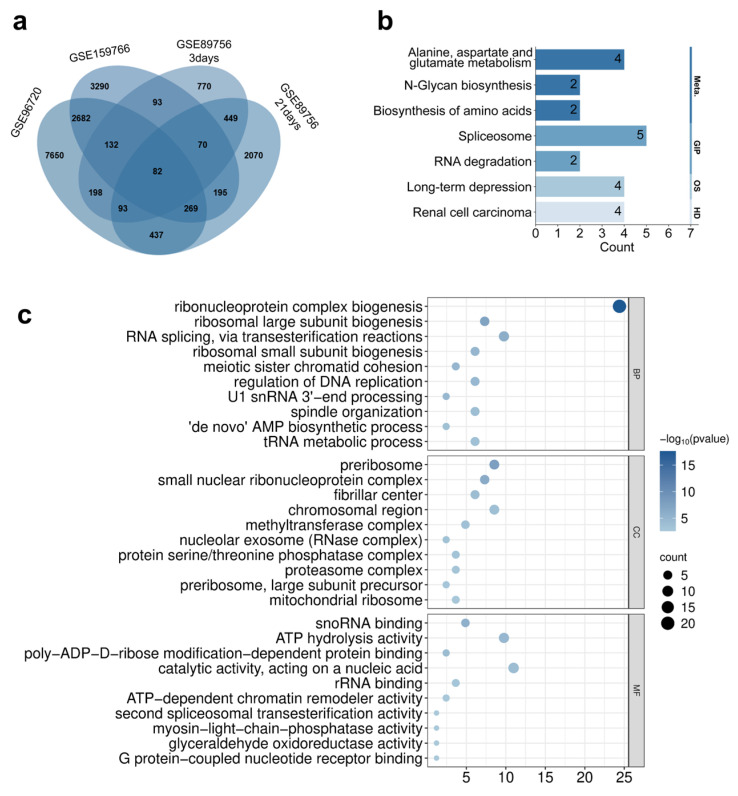
(**a**) Intersection of differentially expressed genes (DEGs) from four GEO datasets; (**b**) KEGG pathway enrichment analysis; and (**c**) GO functional enrichment analysis of the 82 intersected DEGs.

To further integrate transcriptomic evidence, we performed an intersection analysis of the DEGs across the four datasets (Figure 6a). The results revealed that 82 genes exhibited consistent differential expression in all datasets, suggesting that these genes may be key targets for the toxic effects induced by quantum dots. KEGG pathway enrichment analysis was conducted on these 82 intersecting genes (Figure 6b). The results showed that these genes were primarily enriched in pathways such as spliceosome, RNA degradation, biosynthesis of amino acids, alanine, aspartate and glutamate metabolism, long-term depression, and renal cell carcinoma. These pathways are closely related to gene transcription regulation, energy metabolism, and neurological dysfunction.

GO functional annotation analysis (Figure 6c) demonstrated that, in terms of biological processes (BPs), the intersecting genes were significantly enriched in processes such as ribonucleoprotein complex biogenesis, ribosome subunit biogenesis, and RNA splicing. At the cellular component (CC) level, they were mainly associated with structures like the nucleolus, precursor bodies, and proteasome complexes. Regarding molecular functions (MFs), they were concentrated in functions such as RNA binding, ATP hydrolysis activity, and chromatin remodeling. The aforementioned results indicate that these key differentially expressed genes play crucial roles in RNA metabolism, protein synthesis, and cellular stress responses, potentially serving as the molecular basis for cell damage and neurotoxicity induced by quantum dots.

### 3.5. Drug Prediction and Molecular Docking Validation of Core Target Genes

**Figure 7:**
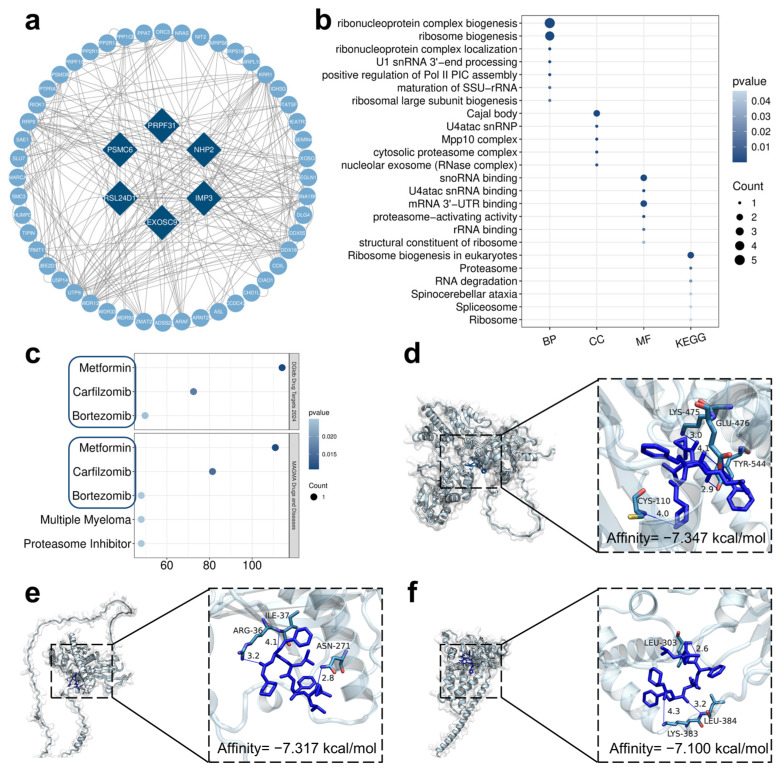
(**a**) Protein–protein interaction (PPI) network of the 82 intersected DEGs. Six core target genes were identified based on network centrality. (**b**) GO and KEGG enrichment analyses of the six core target genes, highlighting their biological functions and pathways. (**c**) Drug target enrichment analysis of the six core genes using DGIdb Drug Targets 2024 and MAGMA Drugs and Diseases datasets. Molecular docking results showing interactions between Carfilzomib and three selected core targets, IMP3 (**d**), EXOSC9 (**e**), and PSMC6 (**f**). Docking scores indicate potential binding affinity.

After performing a protein–protein interaction (PPI) network analysis on the 82 intersecting differentially expressed genes (Figure 7a), six core target genes were identified, including IMP3, EXOSC9, PRPF31, NHP2, RSL24D1, and PSMC6. These core genes exhibited high connectivity within the network, suggesting their pivotal positions in the regulatory network of the intersecting genes. We conducted GO and KEGG enrichment analyses on these core genes, and the results revealed that they were primarily enriched in key biological processes and signaling pathways such as ribosome biogenesis, RNA processing, proteasome function, spliceosome, and RNA degradation (Figure 7b). This indicates that these targets play crucial roles in maintaining post-transcriptional regulation and protein homeostasis in cells.

To explore potential strategies to mitigate QD-induced toxicity, we performed drug target enrichment analysis using DGIdb and MAGMA datasets. This analysis aimed to identify drugs acting on the same targets as the core genes affected by QDs, providing candidates that may counteract the toxic effects of QDs. The results showed that these six potential targets were closely associated with proteasome inhibitors, such as Bortezomib and Carfilzomib (Figure 7c). These findings suggest that proteasome inhibitors may exert their effects by directly or indirectly regulating these core genes.

On this basis, we further conducted molecular docking verification. The docking results demonstrated that the candidate drugs could all form stable binding modes with the core targets (Figure 7d–f, Table S5). Among them, Carfilzomib exhibited the strongest binding affinity, with energy values significantly lower than −7.0 kcal/mol when binding to IMP3 (−7.347 kcal/mol), EXOSC9 (−7.317 kcal/mol), and PSMC6 (−7.100 kcal/mol). Specifically, Carfilzomib formed hydrogen bonds and hydrophobic interactions with amino acids such as GLU-476, TYR-544, and CYS-110 in the active pocket of IMP3; hydrogen bonds with ASN-271 and ARG-361 in the binding cavity of EXOSC9; and stable interactions with residues like LEU-303, LYS-383, and LEU-384 in PSMC6. These interactions enhanced the binding stability between the ligand and the protein. In contrast, Bortezomib exhibited slightly higher binding energies (−6.0 to −6.6 kcal/mol), while Metformin generally showed weaker binding energies (−4.0 to −5.1 kcal/mol). In summary, the database screening and molecular docking results consistently indicated that Carfilzomib had the strongest binding capacity to the core targets, outperforming Bortezomib and Metformin, suggesting that it may be the most promising regulatory molecule acting on the identified targets.

## 4. Discussion

This study systematically integrated machine learning prediction and brain organoid experimental validation, as well as transcriptomics and molecular docking analysis, to construct a multi-module integrated framework of “prediction–validation–intervention” for a systematic exploration of the toxic effects of QDs. The results showed that different types of QDs exhibited significant effects on three key toxic endpoints: cell death, inflammation, and oxidative stress. Moreover, machine learning models, particularly Random Forest (RF) and XGBoost, could stably capture these effects and were highly consistent with the results of brain organoid experiments. This outcome not only validated the application potential of machine learning in nanotoxicology research but also provided new insights for the rapid prediction and mechanism exploration of QD-related toxicity.

Firstly, the comparison results of machine learning models revealed that RF and XGBoost demonstrated the best overall performance in predicting different toxic endpoints, suggesting that these machine learning models could effectively handle the nonlinear and complex relationships between the physicochemical properties of QDs and their toxic effects [44]. This finding not only validated the robustness of machine learning models in small-sample, high-dimensional toxicological data but also offered methodological references for nanomaterial toxicity prediction. Further SHAP analysis revealed that exposure dose and particle size were common key factors across models for different toxic outcomes, while Zeta potential and optical properties exhibited differential effects across endpoints. This discovery indicated that the occurrence of QD toxicity was not driven by a single physicochemical property but rather resulted from a composite effect of multifactorial interactions. Dose and particle size might determine the initial interaction intensity between QDs and biological systems by influencing cell membrane penetration ability, in vivo distribution patterns, and metabolic clearance rates. In contrast, Zeta potential and optical properties could affect downstream oxidative stress and inflammatory pathways by regulating surface reactivity and energy transfer processes [25,45,46,47]. Compared with the prevailing conclusion in previous studies that smaller particle size and higher surface activity lead to stronger toxicity [48,49,50,51,52], this study further clarified the relative weights and action modes of different physicochemical factors in multiple toxic endpoints through model interpretability analysis. This not only enhanced the understanding of QD toxicity mechanisms but also provided ideas for regulating the biocompatibility of QDs through physicochemical parameters in the future.

Additionally, the differences in feature importance ranking among different machine learning models primarily stem from their algorithmic principles, capabilities in capturing feature interactions, and sensitivity to the distribution structure of features [53,54,55]. Although the KNN, RF, and XGBoost models all identify exposure dose and particle size as the core driving factors for quantum dot-induced cytotoxic responses in terms of overall trends, they exhibit significant differences in their emphasis on physicochemical property variables. The KNN model conducts classification based on local distance metrics between samples, relying more on feature clustering and gradient changes in multidimensional space. Consequently, it tends to highlight features such as Zeta potential and hydrodynamic diameter (H_2_O-DLS) that significantly influence colloidal stability and dispersion state, as these factors directly determine the similarity structure among samples [56,57]. The RF model iteratively splits the feature space through multiple random decision trees, selecting variables that maximize information gain. Thus, at a global level, it is more prone to capturing features that can form clear threshold effects, such as exposure dose and transmission electron microscopy particle size (TEM) [58,59,60,61]. The XGBoost model, building upon RF, introduces a gradient boosting strategy to iteratively optimize residuals, thereby capturing high-order nonlinearities and feature interactions. As a result, it further reinforces the dominant roles of exposure dose and particle size (both TEM and H_2_O-DLS) and identifies the synergistic contributions of optical parameters such as emission peak and excitation peak. This suggests that physicochemical properties may participate in regulating toxic effects by modulating energy band structures and electron excitation behaviors [62,63,64,65]. Overall, KNN reflects continuous effects dominated by local similarity, RF emphasizes globally separable discriminative features, and XGBoost integrates complex nonlinearities and interaction effects. The differences among these models do not arise from algorithmic inconsistencies but rather reflect their complementary capabilities in parsing feature spaces. Therefore, multi-model SHAP analysis not only enhances the robustness of the results but also reveals the complex mechanisms by which the physicochemical properties of quantum dots and exposure conditions collectively shape toxicity endpoints such as cell death, inflammation, and oxidative stress at different levels. This provides a more interpretable evidence base for understanding their multidimensional biological responses.

Brain organoid experiments provided direct experimental evidence. CdTe QDs, CQDs, and N-GQDs significantly induced cell damage and oxidative stress in a dose-dependent manner. This finding was similar to early in vitro neurotoxicity studies [12,17,18]. However, compared with two-dimensional cell models and model organisms, brain organoids could more realistically simulate the human neural tissue microenvironment, suggesting that organoids combined with machine learning could more accurately predict the neurotoxicity of QDs. Although there were slight discrepancies between some predicted results of CdTe QDs and experimental data, the overall consistency indicated that the model had good generalization ability across different types of QDs.

Bioinformatics analysis revealed that 82 intersecting differentially expressed genes were mainly enriched in pathways related to RNA metabolism, protein synthesis, and neural function. This was consistent with previous reports that nanomaterials could induce neurotoxicity and cell damage by interfering with RNA processing and energy metabolism [66,67,68,69]. These results also suggested that the toxic effects induced by QDs were not limited to oxidative stress and cell death but might further trigger neurotoxicity and systemic damage by interfering with RNA processing and energy metabolism pathways. The six core targets (IMP3, EXOSC9, PRPF31, NHP2, RSL24D1, PSMC6) screened from the protein–protein interaction (PPI) network indicated that QDs might induce downstream cytotoxic effects by regulating key nodes such as ribosome biogenesis, RNA splicing, and proteasome function. Compared with previous studies on the toxicity mechanisms of nanoparticles [15,18,19,20,21,22], this study provided more systematic molecular evidence across multiple endpoints and offered potential targets for subsequent drug intervention research.

Notably, the results of drug prediction and molecular docking analysis showed that proteasome inhibitors, especially Carfilzomib, had the lowest binding energy with core target genes, demonstrating the strongest binding ability. This suggested that proteasome inhibitors might be a class of potential intervention drugs for the transcriptional regulation and protein homeostasis imbalance induced by QDs. Of course, as an anticancer drug, Carfilzomib itself had strong side effects [70,71,72], so its application in QD toxicity intervention still required further validation and safety assessment. However, this result provided a directional reference for exploring nanomaterial toxicity intervention strategies.

Overall, the multi-module integrated framework established in this study could not only achieve multi-endpoint toxicity prediction of QDs but also reveal potential mechanisms by combining organoid experiments and transcriptomics, as well as explore intervention strategies. Compared with traditional single in vitro or in vivo experiments, this method significantly improved the systematicity and efficiency of toxicity assessment, providing a theoretical and methodological basis for future safety evaluation and intervention strategy development of QDs. However, this study was still limited by the types of QDs and the scale of data. Future research should incorporate more types of QDs and multi-dose, multi-endpoint, and multi-omics data to further enhance the generalization ability and prediction reliability of the models.

## 5. Conclusions

This study established a machine learning-based multi-endpoint toxicity prediction system for QDs, integrating brain organoid experiments, transcriptomic analysis, and molecular docking verification to form an integrated “prediction–validation–intervention” framework. The results demonstrated that different types of quantum dots could significantly induce cell death, inflammation, and oxidative stress, with their toxicity regulated by both dose and particle size. The RF and XGBoost models performed best in the predictions, and SHAP analysis revealed that exposure dose and particle size were the key driving factors. Transcriptomic and molecular docking analyses suggested that QDs might mediate toxicity through RNA metabolism and protein homeostasis, while the proteasome inhibitor Carfilzomib showed potential for intervention. This study not only provides an efficient and systematic approach for evaluating the toxicity of quantum dots but also offers a theoretical foundation and practical reference for future safety management and the development of potential intervention strategies for quantum dots, holding significant scientific and applied value for the biological safety assessment of nanomaterials.

## Data Availability

The data that support the findings of this study are available from the corresponding author upon reasonable request.

## Supplementary Materials

The following supporting information can be downloaded at https://www.mdpi.com/article/10.3390/toxics13110967/s1, Table S1: Machine learning dataset: features and outcomes with types; Table S2: The summary of Physicochemical Characteristics of Experimental Quantum Dots; Table S3: Machine Learning Model Performance for Predicting Quantum Dot Toxicity Based on Physicochemical Properties; Table S4: Comparison of KNN, Random Forest, and XGBoost Predictions with Experimental Results; Table S5: Molecular Docking of Six Proteins (IMP3, EXOSC9, PRPF31, NHP2, RSL24D1, PSMC6) with Bortezomib, Carfilzomib, and Metformin; Figure S1: Mean SHAP values of LR (a), NB (b), SVM (c), and MLP (d) models for predicting quantum dot-induced cell death, inflammation, and oxidative stress; Figure S2: Volcano plots showing differentially expressed genes identified from four GEO datasets; Section S1: Biological rationale of the most promising candidate targets [73,74,75,76,77,78,79,80,81,82,83,84,85,86,87,88,89,90].

## Abbreviations

The following abbreviations are used in this manuscript:

QDs	Quantum Dots
SHAP	Shapley Additive exPlanations
RF	Random Forest
XGBoost	Extreme Gradient Boosting
KNN	K-Nearest Neighbors
SVM	Support Vector Machine
NB	Naive Bayes
LR	Logistic Regression
MLP	Multi-Layer Perceptron
EBs	Embryoid bodies
NIM	Neural induction medium
LDH	Lactate dehydrogenase
MDA	Malondialdehyde
DEGs	Differentially expressed genes
GO	Gene Ontology
KEGG	Kyoto Encyclopedia of Genes and Genomes
PPI	Protein–protein interaction
PLIP	Protein–Ligand Interaction Profiler
