# Supplementary material for: Machine Learning Framework for Multi-Endpoint Quantum Dot Toxicity Prediction with Organoid Validation and Drug Target Discovery
Source: Toxics. 2025 Nov 10;13(11):967. doi: 10.3390/toxics13110967 (PMC12656142; doi:10.3390/toxics13110967)
Supplement: Supplementary file 1 [file toxics-13-00967-s001.zip › toxics-3946678-supplementary.pdf]

Supporting Information

Machine learning framework for multi-endpoint quantum dot toxicity prediction with organoid validation and drug target discovery

Jiafu Yang[1], Pengcheng Xing[1], Siyuan Chen[1], Kehan Liu[1], Zongjian Ye[1], Jieyi Xia[1], Jing He[1], Yijing Qian[1], Dayu Hu[1], and Tianshu Wu[1,*]

[1] *Key Laboratory of Environmental Medicine and Engineering, Ministry of Education; School of Public Health, Southeast University, Nanjing 210009, China.*

[*] Author to whom correspondence should be addressed. E-Mail: ninatswu@seu.edu.cn (T. Wu)

Table S1: Machine learning dataset: features and outcomes with types.

	Features										Outcomes		
**Variable**	Size (nm) in H2O-DLS	Size (nm)-TEM	Excitation peak in H2O (nm)	Emission peak in H2O (nm)	Zeta potential in H2O (mV)	Exposure duration (days)	Exposure dose	Dose unit	Exposure route	Post-exposure period (days)	Cell viability and cell death	Inflammati on	Oxidative stress
**Variable type**	Continuous variables	Continuous variables	Continuous variables	Continuous variables	Continuous variables	Continuous variables	Continuous variables	Categorical variables	Categorical variables	Continuous variables	Categorical variables	Categorical variables	Categorical variables

In this study, data were collected from 102 published reports, and after screening for completeness and quality, 40 studies were included, yielding a total of 306 valid records (out of 646 initially extracted). For machine learning purposes, categorical features were encoded numerically. Specifically, exposure route was encoded as in vitro = 1, intranasal = 2, tail vein = 3, oral = 4, subcutaneous = 5, and dose unit was encoded as μg/mL = 1, μM = 2, mg/kg = 3, μmol/kg = 4. All outcome variables are binary (1 = toxicity, 0 = no toxicity).

Table S2: The Summary of Physicochemical Characteristics of Experimental Quantum Dots.

QDs	Mean size by TEM (nm)	Mean size by DLS in DI water (nm)	ξ-potential in DI water (mV)	Excitation peak in DI water (nm)	Emission peak in DI water (nm)
N-GQDs	3	4.6	-9.9	350	420
CdTe QDs	4.7	4.62	-22.5	380	613
CQDs	3.12	4.73	-2.48	380	387

Table S3: Machine Learning Model Performance for Predicting Quantum Dot Toxicity Based on Physicochemical Properties.

Outcome	Modle	LR	RF	KNN	SVM	XGBoost	NB	MLP
Cell viability and cell death	Accuracy	0.73913	0.836957	0.804348	0.771739	0.815217	0.521739	0.695652
	Sensitivity	0.941176	0.862745	0.784314	0.764706	0.843137	0.196078	0.745098
	Specificity	0.487805	0.804878	0.829268	0.780488	0.780488	0.926829	0.634146
	Precision	0.695652	0.846154	0.851064	0.8125	0.826923	0.769231	0.716981
	F1 score	0.8	0.854369	0.816327	0.787879	0.834951	0.3125	0.730769
	ROC-AUC	0.723577	0.928025	0.863223	0.813486	0.902917	0.702056	0.736011
	PR-AUC	0.748488	0.946722	0.899786	0.83831	0.926156	0.721057	0.770769
Inflammation	Accuracy	0.836957	0.891304	0.891304	0.902174	0.913043	0.336957	0.880435
	Sensitivity	1	0.974026	0.961039	1	0.974026	0.220779	0.974026
	Specificity	0	0.466667	0.533333	0.4	0.6	0.933333	0.4
	Precision	0.836957	0.903614	0.91358	0.895349	0.925926	0.944444	0.892857
	F1 score	0.911243	0.9375	0.936709	0.944785	0.949367	0.357895	0.931677
	ROC-AUC	0.708225	0.978355	0.8	0.805195	0.956277	0.7671	0.833766
	PR-AUC	0.90281	0.996057	0.950989	0.946322	0.991238	0.937604	0.930455
Oxidative stress	Accuracy	0.847826	0.902174	0.880435	0.858696	0.902174	0.304348	0.847826
	Sensitivity	0.986667	0.986667	0.986667	0.986667	1	0.16	0.946667
	Specificity	0.235294	0.529412	0.411765	0.294118	0.470588	0.941176	0.411765
	Precision	0.850575	0.902439	0.880952	0.860465	0.892857	0.923077	0.876543
	F1 score	0.91358	0.942675	0.930818	0.919255	0.943396	0.272727	0.910256
	ROC-AUC	0.774902	0.897647	0.807843	0.722353	0.904706	0.700784	0.786667
	PR-AUC	0.904323	0.966044	0.924544	0.888595	0.961663	0.902352	0.90514

Table S4: Comparison of KNN, Random Forest, and XGBoost Predictions with Experimental Results.

QDs	KNN				RF				XGBoost			
	Cell viability and cell death		Oxidative stress		Cell viability and cell death		Oxidative stress		Cell viability and cell death		Oxidative stress	
	Prediction	Experiment	Prediction	Experiment	Prediction	Experiment	Prediction	Experiment	Prediction	Experiment	Prediction	Experiment
**CQDs-1**	1	1	1	1	1	1	1	1	1	1	1	1
**CQDs-2**	1	1	1	1	1	1	1	1	1	1	1	1
**CQDs-3**	1	1	1	1	1	1	1	1	1	1	1	1
**N-GQDs-1**	1	1	1	1	1	1	1	1	1	1	1	1
**N-GQDs-2**	1	1	1	1	1	1	1	1	1	1	1	1
**N-GQDs-3**	1	1	1	1	1	1	1	1	1	1	1	1
**CdTe QDs-1**	0	1	1	1	0	1	0	1	0	1	0	1
**CdTe QDs-2**	0	1	1	1	0	1	0	1	0	1	0	1
**CdTe QDs-3**	0	1	1	1	0	1	1	1	0	1	1	1

1 indicates the occurrence of toxicity; 0 indicates no toxicity.

Table S5: Molecular Docking of Six Proteins (IMP3, EXOSC9, PRPF31, NHP2, RSL24D1, PSMC6) with Bortezomib, Carfilzomib, and Metformin.

Ligand		Receptor					
		IMP3	EXOSC9	PRPF31	NHP2	RSL24D1	PSMC6
Affinity (kcal/mol)	Bortezomib	-6.063	-6.062	-6.004	-6.321	-6.521	-6.277
	Carfilzomib	-7.347	-7.317	-6.666	-6.993	-6.943	-7.100
	Metformin	-4.212	-5.193	-4.335	-4.497	-4.000	-4.582

Affinity represents the binding affinity (ΔG, kcal/mol) between the ligand and the protein. More negative values indicate stronger and more stable binding.

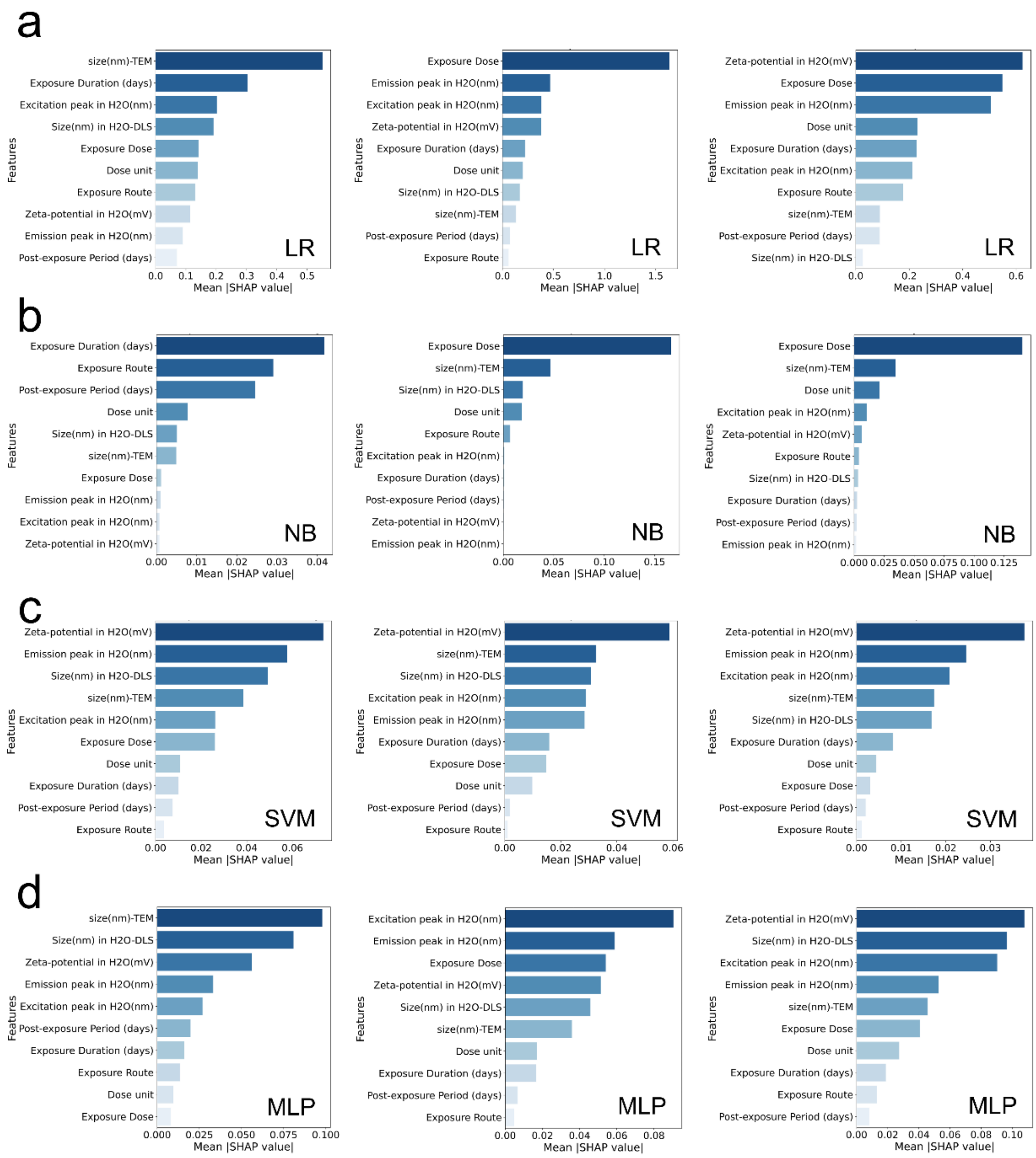

Figure S1: Mean SHAP values of LR (a), NB (b), SVM (c), and MLP (d) models for predicting quantum dot-induced cell death, inflammation, and oxidative stress.

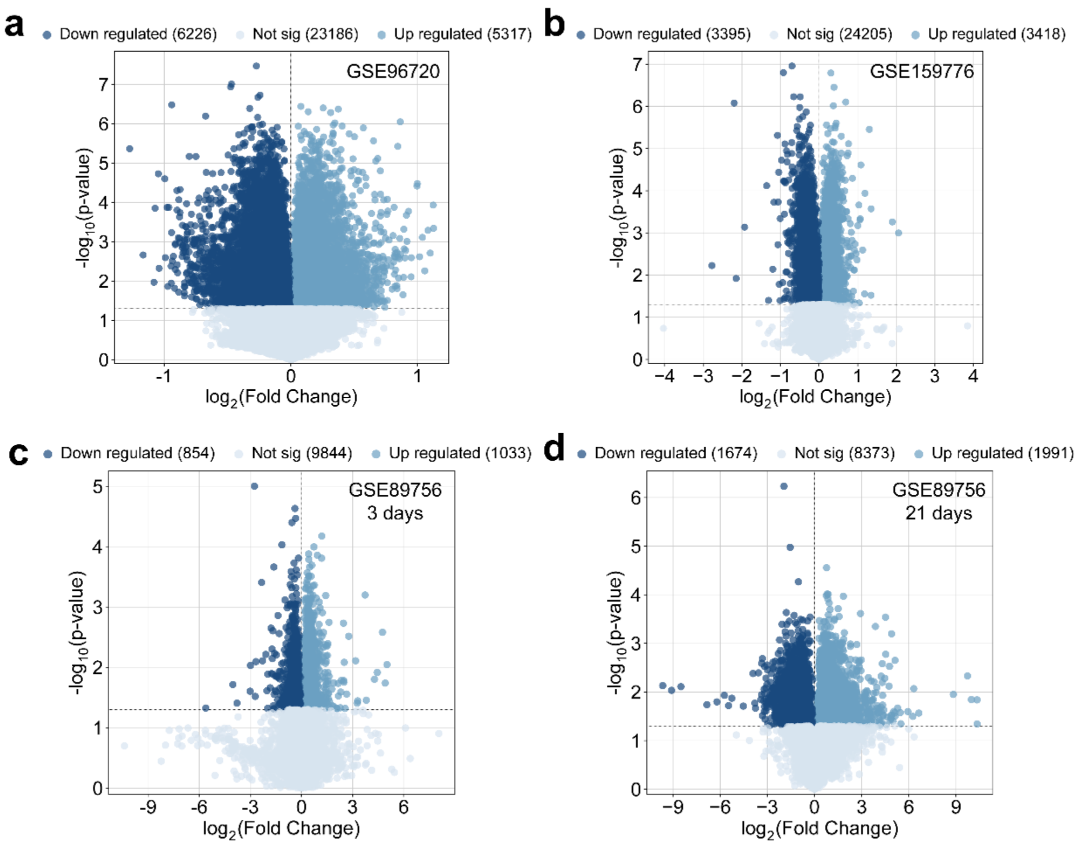

Figure S2: Volcano plots showing differentially expressed genes identified from four GEO datasets.

Section S1: Biological rationale of the most promising candidate targets.

Based on our network pharmacology analysis, six candidate targets (IMP3, EXOSC9, PRPF31, NHP2, RSL24D1, and PSMC6) have been identified as potentially being associated with the toxicity induced by quantum dots (QDs). Existing literature has elucidated the biological roles of these targets, as well as their correlations with cellular stress responses, RNA metabolism, and protein homeostasis.

IMP3 (IGF2BP3) is an RNA-binding protein that regulates the stability and translation of messenger ribonucleic acid (mRNA), thereby influencing cell proliferation and survival under stress conditions. Its dysfunctional regulation is linked to altered stress responses and impaired neuronal function [73-75]. EXOSC9, a core component of the RNA exosome complex, is involved in RNA processing and degradation, and its functional abnormalities are associated with neurodegenerative phenotypes [76-78]. PRPF31 participates in the splicing process of precursor messenger ribonucleic acid (pre-mRNA) and regulates cellular homeostasis. It is known that mutations in PRPF31 can sensitize cells to stress [79,80]. NHP2 is crucial for the assembly of ribonucleoprotein complexes and telomere maintenance, both of which play a pivotal role in maintaining genomic stability under oxidative or toxic stress conditions [81-83]. RSL24D1 plays a key role in ribosome biogenesis, and its functional abnormalities can disrupt protein synthesis and trigger cellular stress responses [84-87]. PSMC6, a subunit of the 26S proteasome, is involved in protein degradation and quality control processes, preventing the accumulation of misfolded or damaged proteins and thereby avoiding exacerbation of cellular toxicity [88-90].

Overall, these targets are mechanistically linked to multiple pathways, including RNA metabolism, ribosome function, protein homeostasis, and stress response signaling pathways, which may be modulated during QD exposure. The molecular docking analysis conducted in this study provides preliminary predictions of possible interactions between QDs and these targets, aiding in the identification of candidate targets for future experimental validation. Further in vitro and in vivo experimental studies are still required to confirm the functional relevance of these targets and to evaluate the potential pharmacological or toxicological effects of QDs on them.
